# Development and Validation of a Prediction Model for Acute Coronary Occlusion in Patients With Non‐ST‐Segment Elevation Myocardial Infarction

**DOI:** 10.1002/clc.70492

**Published:** 2026-10-22

**Authors:** Qicheng Yu, Shuai Wang, Yuanyuan Zhao, Yanlong Zhao, Shan Xie, Yanling Wang, Zhenxing Fan, Fangyan Liu, Zhi Liu

**Affiliations:** ^1^ Department of Emergency, Xuanwu Hospital, Capital Medical University National Clinical Research Center for Geriatric Diseases Beijing China; ^2^ Department of Cardiology Xuanwu Hospital, Capital Medical University Beijing China; ^3^ Department of Geriatrics, Xuanwu Hospital Capital Medical University Beijing China

**Keywords:** acute coronary occlusion, nomogram, non‐ST‐segment elevation myocardial infarction, prediction model

## Abstract

**Background:**

Acute coronary occlusion (ACO) occurs in approximately one‐quarter of patients with non‐ST‐segment elevation myocardial infarction (NSTEMI) and is associated with adverse outcomes. This study aimed to develop and validate a prediction model for ACO.

**Methods:**

Consecutive patients admitted with NSTEMI between January 2016 and June 2023 who underwent coronary angiography were enrolled. Demographic, electrocardiographic, laboratory, and echocardiographic data were collected. Patients admitted during 2016−2020 were assigned to the training cohort (*n* = 670), and those admitted during 2021−2023 were assigned to the validation cohort (*n* = 334). Predictors were selected using least absolute shrinkage and selection operator regression, and a nomogram was constructed based on multivariable logistic regression. Model performance was assessed by receiver operating characteristic (ROC) curves, calibration curves, and decision curve analysis (DCA).

**Results:**

Among 1,004 patients, 262 (26.1%) had ACO. Seven predictors, including smoking history, ST‐segment depression ≥ 1 mm, regional wall motion abnormality, left ventricular ejection fraction, admission cardiac troponin I, interleukin‐6, and high‐sensitivity C‐reactive protein, were incorporated into the model. The area under the ROC curve was 0.731 (95% CI, 0.688−0.773) in the training cohort and 0.715 (95% CI, 0.654−0.776) in the validation cohort. Calibration curves showed good agreement between predicted and observed probabilities, and DCA demonstrated favorable clinical utility.

**Conclusion:**

This study developed and validated a multidimensional prediction model for ACO in patients with NSTEMI. The model showed acceptable performance and may aid early identification before coronary angiography, although reliance on IL‐6, which is not routinely available in all centers, may limit its generalizability.

## Graphical abstract

A multidimensional nomogram incorporating clinical, electrocardiographic, echocardiographic, myocardial injury, and inflammatory markers may help identify NSTEMI patients at high risk of acute coronary occlusion before angiography.

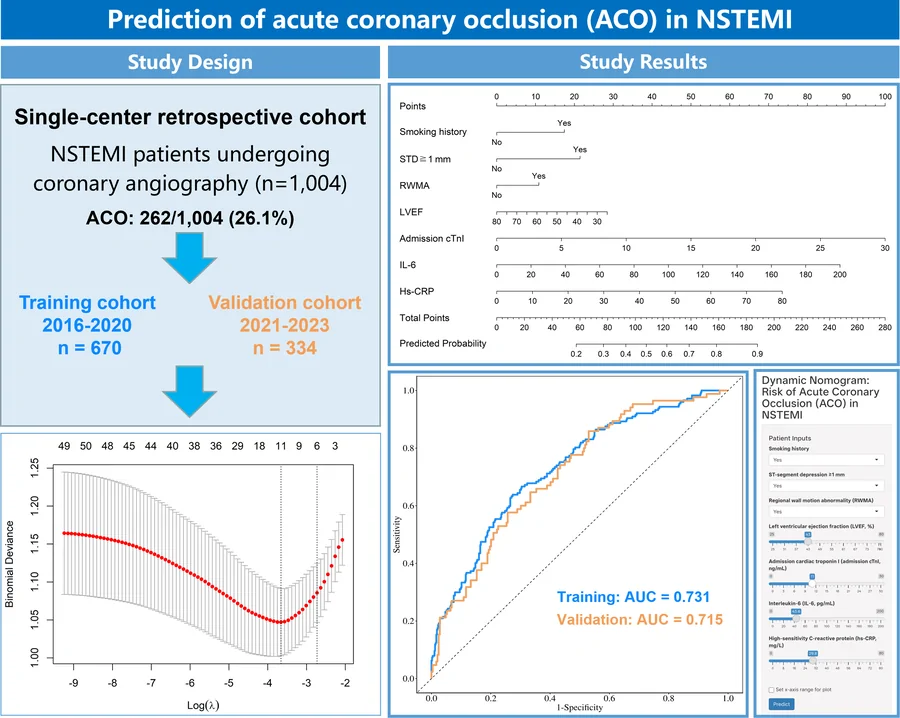

**Abbreviations**
ACO: acute coronary occlusion
AI‐ECG: artificial‐intelligence‐enhanced electrocardiography
DCA: decision curve analysis
Hcy: homocysteine
NLR: neutrophil‐to‐lymphocyte ratio
OMI: occlusive myocardial infarction
RWMA: regional wall motion abnormality
SHR: stress hyperglycemia ratio
STD: ST‐segment depression
VIF: variance inflation factor
α‐HBDH: α‐hydroxybutyrate dehydrogenase

## 1 Introduction

Cardiovascular diseases remain the leading cause of death globally, with acute myocardial infarction (AMI) representing a major contributor and accounting for millions of deaths each year [1]. With the widespread adoption of high‐sensitivity cardiac troponin assays [2, 3] and the progressive aging of the population [4], the diagnosis of non‐ST‐segment elevation myocardial infarction (NSTEMI) has risen substantially [5, 6, 7, 8]. Several studies have indicated that NSTEMI accounts for nearly two‐thirds of all AMI cases [9, 10]. Traditionally, ST‐segment elevation myocardial infarction (STEMI) has been attributed to acute coronary occlusion (ACO), whereas NSTEMI has been considered a consequence of partial or incomplete occlusion [11]. However, two large meta‐analyses demonstrated that approximately 25%−34% of NSTEMI patients present with ACO, and these individuals experience significantly worse outcomes compared with those without ACO [12, 13]. Unlike STEMI patients, who typically undergo reperfusion within hours of symptom onset, NSTEMI patients with ACO often experience delays of 24−48 h or longer before revascularization [12, 14], which may increase the extent of myocardial necrosis and reduce salvageable myocardium. Although advanced modalities such as speckle‐tracking echocardiography and artificial intelligence‐enhanced electrocardiography (AI‐ECG) have shown promise for early detection of occlusive myocardial infarction (OMI) [15, 16, 17, 18], their dependence on specialized equipment, proprietary algorithms, and trained personnel limits widespread implementation. At present, few practical tools are available that leverage multidimensional clinical variables obtained during early hospitalization to predict ACO in NSTEMI patients prior to coronary angiography. Therefore, this study sought to develop and validate a prediction model integrating conventional clinical data, electrocardiographic features, laboratory biomarkers, and echocardiographic parameters, with the goal of supporting the early identification of patients at high risk of ACO before coronary angiography.

## 2 Methods

### 2.1 Study Design and Population

We retrospectively reviewed the electronic medical records of 1,126 patients with NSTEMI who were admitted to the Department of Cardiology, Xuanwu Hospital, Capital Medical University, between January 2016 and June 2023 and underwent coronary angiography. Eligible patients were required to meet the diagnostic criteria for NSTEMI [19] and be aged ≥ 18 years. Patients were excluded if they had a history of coronary artery bypass grafting (CABG), a diagnosis of myocardial infarction with non‐obstructive coronary arteries (MINOCA), an estimated glomerular filtration rate (eGFR) < 30 mL/min/1.73 m^2^, recent infection, autoimmune disease, or hematologic disorder. Patients admitted between January 2016 and December 2020 were assigned to the training cohort, whereas those admitted between January 2021 and June 2023 were assigned to the validation cohort. The patient selection process is illustrated in Figure 1. We prespecified that the model would include no more than 10 predictor parameters. Based on the commonly recommended rule of at least 10 events per variable (EPV) [20, 21], a minimum of 100 events would be required. Given the anticipated prevalence of ACO of approximately 25% [12, 13], this translated into a required sample size of approximately 400 patients. The training cohort included 670 patients with 177 ACO events (EPV = 17.7), thus exceeding this requirement. All data were obtained from Xuanwu Hospital and anonymized prior to analysis. The study protocol was approved by the Ethics Committee of Xuanwu Hospital, Capital Medical University (Approval No. KS2022161‐1), and the requirement for informed consent was waived because of the retrospective design and use of de‐identified data. The study was conducted in accordance with the Declaration of Helsinki. During the study period, invasive management of patients with NSTEMI was guided by contemporary guideline recommendations and individualized clinical judgment.

**Figure 1:**
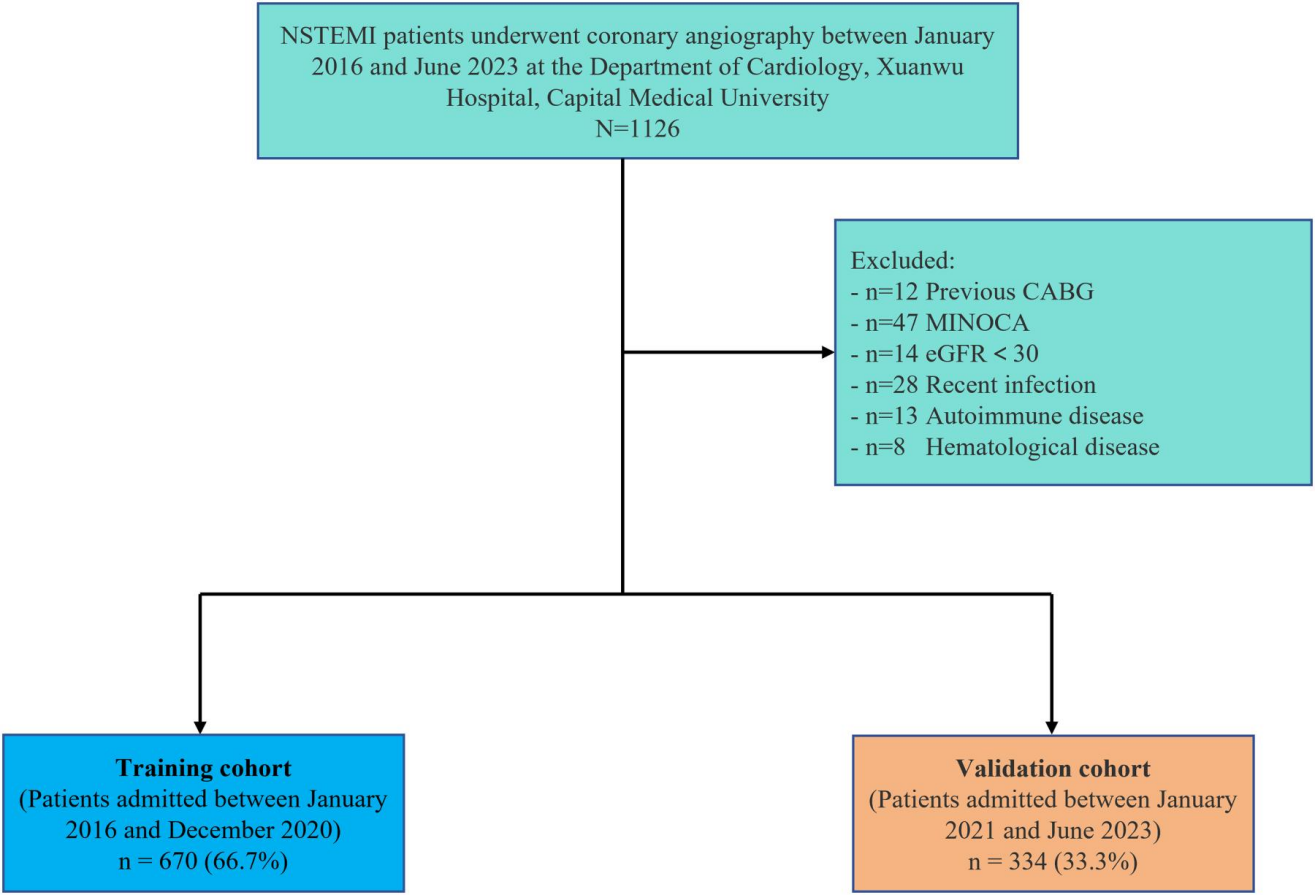
Study flowchart. CABG, coronary artery bypass grafting; eGFR, estimated glomerular filtration rate; MINOCA, myocardial infarction with non‐obstructive coronary arteries; NSTEMI, non‐ST‐segment elevation myocardial infarction.

### 2.2 Data Collection

Demographic and clinical characteristics were collected, including age, sex, body mass index (BMI), history of hypertension and diabetes, as well as smoking history and alcohol consumption status. A standard 12‐lead electrocardiogram (ECG) was obtained on admission using routine clinical settings, with a paper speed of 25 mm/s and calibration of 10 mm/mV. The archived admission ECG tracings were retrospectively and independently re‐reviewed by two senior attending cardiologists who were blinded to the coronary angiography findings. Interobserver agreement between the two cardiologists for the classification of STD ≥ 1 mm was assessed using Cohen's κ coefficient with a 95% confidence interval. Disagreements were resolved by consensus or, when necessary, by consultation with a third senior cardiologist. ST‐segment depression (STD) was defined as horizontal or downsloping STD ≥ 1 mm in at least two contiguous leads, according to standard ECG criteria, and the involved leads were recorded. STD was further classified according to lead distribution: anterior precordial STD was defined as involvement of leads V1−V4, inferior STD as involvement of leads II, III, or aVF, and lateral STD as involvement of leads I, aVL, V5, or V6. Multiterritory STD was defined as involvement of two or more lead territories. Because some patients had STD in more than one territory, these lead‐distribution categories were not mutually exclusive. Echocardiographic data, including left ventricular ejection fraction (LVEF) and regional wall motion abnormality (RWMA), were collected from transthoracic echocardiography performed during the index hospitalization, typically within 24 h of admission. All examinations were performed by experienced sonographers and reviewed by attending cardiologists in routine clinical practice. Fasting peripheral venous blood samples were obtained on the day of admission or the following morning to measure complete blood counts, interleukin‐6 (IL‐6), high‐sensitivity C‐reactive protein (hs‐CRP), total cholesterol (TC), triglycerides (TG), aspartate aminotransferase (AST), and fasting blood glucose (FBG). Creatine kinase‐MB (CK‐MB) and cardiac troponin I (cTnI) were measured every 8 h in four serial assessments. The first cTnI value obtained at admission was defined as admission cTnI, whereas the highest value during serial testing was defined as peak cTnI. The target outcome was ACO, defined as Thrombolysis in Myocardial Infarction (TIMI) flow grade 0 or 1 in the culprit vessel on the first coronary angiography performed during the index hospitalization [22]. All angiograms were interpreted by experienced interventional cardiologists at the time of the procedure, following routine clinical practice. MINOCA was defined as angiographic stenosis < 50% in all major epicardial vessels, in conjunction with clinical presentation and biomarker evidence consistent with myocardial infarction [23]. Smoking history was defined as regular tobacco use (current or former) of at least one cigarette per day for ≥ 6 months, whereas non‐smokers were defined as individuals with no such history or with a cumulative smoking duration < 6 months. The neutrophil‐to‐lymphocyte ratio (NLR) was calculated as the neutrophil count divided by the lymphocyte count obtained from the admission blood sample. The stress hyperglycemia ratio (SHR) was defined as the ratio of baseline FBG (mmol/L) to the estimated average glucose derived from glycated hemoglobin (HbA1c), calculated as: FBG/(1.59 × HbA1c [%]–2.59) [24, 25].

### 2.3 Statistical Analysis

The distribution of continuous variables was assessed using the Kolmogorov‐Smirnov test. Normally distributed variables were expressed as mean ± standard deviation and compared using the independent‐samples *t* test, whereas non‐normally distributed variables were presented as median (interquartile range, IQR) and compared using the Mann−Whitney *U* test. Categorical variables were reported as counts (percentages) and compared using the chi‐square test or Fisher's exact test. The missing proportion of each candidate predictor was assessed before imputation. A prespecified threshold of 20% missingness was applied as a data‐quality criterion to avoid unstable imputation of variables with substantial missingness. All candidate predictors had missing proportions below this threshold; therefore, no candidate predictor was excluded because of excessive missingness. Missing values were imputed separately in the training and validation cohorts using the random forest algorithm [26]. The proportions of missing data for each variable are shown in Supporting Information S1: Tables 1 and 2. In the training cohort, 50 candidate predictors were identified based on prior literature and clinical relevance (Supporting Information: Appendix). Variable selection was performed using the least absolute shrinkage and selection operator (LASSO) regression, with the optimal penalty parameter (λ) determined by tenfold cross‐validation to reduce the risk of overfitting. To develop a parsimonious and clinically interpretable model, variables selected by LASSO were further assessed for collinearity and redundancy using the variance inflation factor (VIF) and Spearman correlation analysis. Predefined criteria were used, including a VIF > 5 or an absolute Spearman correlation coefficient > 0.70. When highly correlated variables reflected overlapping clinical or biological information, one variable was retained based on clinical interpretability, temporal availability, and measurement reliability. The remaining variables were subsequently entered into a multivariable logistic regression model, and a nomogram was constructed for visualization. Predictors retained in the final model were determined by the prespecified LASSO‐based selection procedure and subsequent assessment of collinearity and redundancy, rather than by post hoc elimination based on individual Wald *P* values in the multivariable logistic regression model. Model performance was comprehensively evaluated in terms of discrimination, calibration, and clinical utility. Discriminatory ability was assessed using the receiver operating characteristic (ROC) curve and the area under the curve (AUC). The optimal cutoff value was determined by Youden's index in the training cohort and then applied to both cohorts to generate confusion matrices, from which sensitivity, specificity, positive predictive value (PPV), negative predictive value (NPV), and accuracy were calculated. Calibration was evaluated using calibration curves and the Hosmer–Lemeshow goodness‐of‐fit test, and clinical utility was examined using decision curve analysis (DCA). To evaluate potential overfitting of the final logistic regression model, bootstrap internal validation was performed in the training cohort with 1,000 resamples. The apparent model performance was compared with bootstrap‐corrected estimates to quantify optimism. Optimism‐corrected discrimination was assessed using the area under the ROC curve, and model calibration was evaluated using the calibration slope and Brier score. All statistical analyses were performed using R software, version 4.4.3. A two‐sided *p* value < 0.05 was considered statistically significant.

## 3 Results

### 3.1 Patient Characteristics

A total of 1004 patients were included in the final analysis, with 670 assigned to the training cohort and 334 to the validation cohort. Baseline demographic and clinical characteristics of the two cohorts are summarized in Supporting Information S1: Table 3. The prevalence of ACO was similar between the training and validation cohorts (26.4% vs. 25.4%; *p* = 0.742). Baseline characteristics of patients with and without ACO in the training cohort are shown in Table 1. There were no significant differences in age or sex distribution between the ACO and non‐ACO groups (*p* > 0.05). Compared with the non‐ACO group, patients with ACO had lower admission systolic blood pressure (SBP) (130.0 [119.0−145.0] mmHg vs. 135.0 [123.0−148.0] mmHg; *p* = 0.022) and a higher prevalence of smoking history (67.2% vs. 58.8%; *p* = 0.049). Regarding biomarkers, levels of myocardial injury and inflammatory markers, including admission CK‐MB, admission cTnI, IL‐6, and hs‐CRP, were significantly elevated in the ACO group (all *p* < 0.05). With respect to cardiac function, patients with ACO exhibited higher N‐terminal pro‐B‐type natriuretic peptide (NT‐proBNP) levels (625.0 [266.7−1485.0] pg/mL vs. 464.0 [178.0−1166.0] pg/mL; *p* = 0.016), lower LVEF (59.0 [53.0−64.0]% vs. 62.0 [56.0−67.0]%; *p* < 0.001), and a greater prevalence of RWMA (74.6% vs. 60.9%; *p* = 0.001). On electrocardiography, the proportion of patients with STD ≥ 1 mm was higher in the ACO group (63.3% vs. 44.6%; *p* < 0.001), whereas the frequency of T‐wave inversion did not differ between groups (42.4% vs. 38.3%; *p* = 0.346). In an exploratory lead‐distribution analysis, anterior precordial STD, inferior STD, lateral STD, and multiterritory STD were all more frequently observed in patients with ACO than in those without ACO (Supporting Information S1: Table 4). The two cardiologists agreed on the classification of STD ≥ 1 mm in 964 of 1004 patients (96.0%), with a Cohen's κ coefficient of 0.918 (95% CI, 0.892–0.942) (Supporting Information S1: Table 5).

**Table 1:** Comparative analysis of variables between patients with ACO and patients with non‐ACO in the training cohort.

Variables	Total (*n* = 670)	Non‐ACO (*n* = 493)	ACO (*n* = 177)	*p*
Age (years)	62.00 (56.00, 69.75)	63.00 (57.00, 70.00)	62.00 (55.00, 69.00)	0.354
Sex (Male), *n* (%)	516 (77.0)	375 (76.1)	141 (79.7)	0.329
Heart rate (bpm)	74.00 (66.00, 83.00)	74.00 (66.00, 82.00)	74.00 (64.00, 84.00)	0.892
BMI (kg/m^2^)	25.76 (23.94, 27.77)	25.71 (24.09, 27.68)	25.89 (23.66, 28.34)	0.915
SBP (mmHg)	134.00 (122.00, 147.75)	135.00 (123.00, 148.00)	130.00 (119.00, 145.00)	0.022
DBP (mmHg)	75.00 (67.00, 84.00)	75.00 (67.00, 85.00)	75.00 (67.00, 83.00)	0.687
Smoking history, *n* (%)	409 (61.0)	290 (58.8)	119 (67.2)	0.049
Alcohol use, *n* (%)	268 (40.0)	203 (41.2)	65 (36.7)	0.300
Hypertension, *n* (%)	438 (65.4)	320 (64.9)	118 (66.7)	0.673
DM, *n* (%)	273 (40.7)	204 (41.4)	69 (39.0)	0.578
Previous MI, *n* (%)	60 (9.0)	49 (9.9)	11 (6.2)	0.137
Previous PCI, *n* (%)	76 (11.3)	59 (12.0)	17 (9.6)	0.395
Previous stroke, *n* (%)	92 (13.7)	69 (14.0)	23 (13.0)	0.740
CK (IU/L)	148.50 (83.25, 333.50)	128.00 (78.00, 254.00)	256.00 (112.00, 649.00)	< 0.001
Myoglobin (ng/mL)	44.00 (22.10, 96.30)	40.00 (20.90, 86.70)	60.70 (25.60, 151.00)	< 0.001
α‐HBDH (IU/L)	181.00 (141.00, 269.00)	173.00 (135.00, 237.00)	241.00 (165.00, 368.00)	< 0.001
LDH (IU/L)	221.00 (178.00, 305.00)	212.00 (172.00, 274.00)	269.00 (199.00, 412.00)	< 0.001
AST (IU/L)	31.00 (22.00, 55.00)	29.00 (21.00, 44.00)	47.00 (26.00, 78.00)	< 0.001
Admission CK‐MB (ng/mL)	8.54 (3.25, 25.00)	6.69 (2.80, 19.10)	18.80 (5.97, 52.00)	< 0.001
Admission cTnI (ng/mL)	0.29 (0.09, 1.10)	0.23 (0.07, 0.77)	0.72 (0.16, 2.15)	< 0.001
Peak CK‐MB (ng/mL)	16.65 (5.13, 58.00)	11.10 (4.17, 38.60)	47.20 (11.00, 125.00)	< 0.001
Peak cTnI (ng/mL)	1.12 (0.31, 4.36)	0.79 (0.26, 2.88)	3.69 (0.82, 9.16)	< 0.001
NT‐proBNP (pg/mL)	500.00 (189.00, 1242.25)	464.00 (178.00, 1166.00)	625.00 (266.70, 1485.00)	0.016
IL‐6 (pg/mL)	19.16 (9.42, 32.82)	16.70 (8.06, 28.83)	28.64 (16.36, 48.50)	< 0.001
Hs‐CRP (mg/L)	6.93 (2.30, 16.17)	5.97 (1.94, 13.57)	11.99 (4.74, 29.48)	< 0.001
Albumin (g/L)	37.14 (34.96, 39.40)	37.32 (35.00, 39.40)	36.65 (34.85, 39.29)	0.279
eGFR (mL/min/1.73 m^2^)	100.19 (90.56, 106.16)	100.39 (90.49, 105.94)	99.83 (90.67, 107.09)	0.986
Creatinine (μmol/L)	70.00 (61.00, 81.00)	69.00 (61.00, 81.00)	70.00 (61.00, 81.00)	0.543
Uric acid (μmol/L)	340.00 (279.25, 403.75)	339.00 (285.00, 406.00)	342.00 (275.00, 399.00)	0.833
HDL‐C (mmol/L)	0.98 (0.85, 1.17)	0.98 (0.84, 1.18)	0.98 (0.86, 1.14)	0.734
LDL‐C (mmol/L)	2.54 (2.04, 3.09)	2.51 (1.97, 3.07)	2.61 (2.15, 3.15)	0.080
Apolipoprotein A1 (g/L)	1.09 (0.97, 1.22)	1.10 (0.97, 1.24)	1.07 (0.97, 1.19)	0.093
Apolipoprotein B (g/L)	0.87 (0.74, 1.04)	0.87 (0.74, 1.04)	0.89 (0.75, 1.05)	0.222
Triglyceride (mmol/L)	1.65 (1.16, 2.31)	1.66 (1.19, 2.27)	1.59 (1.07, 2.37)	0.466
Total cholesterol (mmol/L)	4.17 (3.57, 4.88)	4.11 (3.53, 4.87)	4.26 (3.72, 4.93)	0.101
Hcy (μmol/L)	14.62 (11.60, 18.40)	14.50 (11.40, 18.36)	14.78 (11.90, 18.50)	0.518
FBG (mmol/L)	6.08 (5.06, 7.92)	6.01 (5.05, 7.93)	6.23 (5.11, 7.81)	0.676
Glycated hemoglobin (%)	6.20 (5.62, 7.50)	6.20 (5.70, 7.50)	6.20 (5.60, 7.50)	0.947
SHR	0.83 (0.74, 0.95)	0.83 (0.74, 0.94)	0.84 (0.74, 0.96)	0.381
TSH (μIU/mL)	1.42 (0.90, 2.25)	1.50 (0.94, 2.27)	1.27 (0.76, 2.16)	0.012
T3 (ng/mL)	0.94 (0.80, 1.04)	0.95 (0.81, 1.04)	0.89 (0.77, 1.00)	0.002
FT3 (pg/mL)	2.66 (2.40, 2.91)	2.68 (2.43, 2.94)	2.59 (2.34, 2.81)	0.003
FT4 (ng/dL)	1.12 (1.01, 1.25)	1.12 (1.02, 1.25)	1.11 (0.99, 1.25)	0.393
NLR	2.88 (2.10, 4.06)	2.71 (2.03, 3.71)	3.33 (2.34, 4.63)	< 0.001
White blood cells (×10^9^/L)	8.02 (6.67, 9.70)	7.76 (6.55, 9.47)	8.88 (7.23, 10.55)	< 0.001
Monocytes (×10^9^/L)	0.46 (0.36, 0.59)	0.45 (0.36, 0.57)	0.48 (0.38, 0.65)	0.009
Neutrophils (×10^9^/L)	5.46 (4.26, 6.75)	5.27 (4.10, 6.51)	6.18 (4.81, 7.68)	< 0.001
Lymphocytes (×10^9^/L)	1.88 (1.44, 2.34)	1.86 (1.46, 2.36)	1.89 (1.40, 2.34)	0.456
Hemoglobin (g/L)	138.00 (126.00, 147.00)	138.00 (126.00, 147.00)	137.00 (126.00, 148.00)	0.767
Platelets (×10^9^/L)	215.00 (175.00, 257.75)	218.00 (176.00, 259.00)	212.00 (171.00, 249.00)	0.173
Fibrinogen (g/L)	3.55 (3.05, 4.16)	3.53 (3.04, 4.08)	3.64 (3.10, 4.33)	0.103
d‐dimer (μg/mL)	0.30 (0.21, 0.47)	0.30 (0.21, 0.46)	0.30 (0.23, 0.49)	0.635
LVEF (%)	61.0 (55.0, 67.0)	62.0 (56.0, 67.0)	59.0 (53.0, 64.0)	< 0.001
RWMA, *n* (%)	432 (64.5)	300 (60.9)	132 (74.6)	0.001
Killip ≥ 2, *n* (%)	194 (29.0)	135 (27.4)	59 (33.3)	0.134
GRACE score (points)	136.00 (113.00, 155.00)	134.00 (112.00, 154.00)	139.00 (119.00, 156.00)	0.064
ST‐segment depression ≥ 1 mm, *n* (%)	332 (49.6)	220 (44.6)	112 (63.3)	< 0.001
T wave inversion, *n* (%)	264 (39.4)	189 (38.3)	75 (42.4)	0.346

Abbreviations: ACO, acute coronary occlusion; AST, aspartate aminotransferase; α‐HBDH, α‐hydroxybutyrate dehydrogenase; BMI, body mass index; CK, creatine kinase; CK‐MB, creatine kinase‐MB isoenzyme; cTnI, cardiac troponin I; DBP, diastolic blood pressure; DM, diabetes mellitus; eGFR, estimated glomerular filtration rate; FBG, fasting blood glucose; FT3, free triiodothyronine; FT4, free thyroxine; GRACE, Global Registry of Acute Coronary Events; Hcy, homocysteine; HDL‐C, high‐density lipoprotein cholesterol; Hs‐CRP, high‐sensitivity C‐reactive protein; IL‐6, interleukin‐6; LDH, lactate dehydrogenase; LDL‐C, low‐density lipoprotein cholesterol; LVEF, left ventricular ejection fraction; MI, myocardial infarction; NLR, neutrophil‐to‐lymphocyte ratio; NT‐proBNP, N‐terminal pro‐B‐type natriuretic peptide; PCI, percutaneous coronary intervention; RWMA, regional wall motion abnormality; SBP, systolic blood pressure; SHR, stress hyperglycemia ratio; T3, triiodothyronine; TSH, thyroid stimulating hormone.

### 3.2 Predictor Selection

Based on prior literature and clinical relevance, 50 candidate variables were initially entered into the LASSO regression. The cross‐validation curve and coefficient profile plot are presented in Figure 2. Using the λ value that minimized cross‐validation error (lambda.min), 11 variables with nonzero coefficients were selected: smoking history, STD ≥ 1 mm, RWMA, LVEF, CK, AST, admission cTnI, peak cTnI, peak CK‐MB, IL‐6, and hs‐CRP. To improve model parsimony and reduce redundancy among closely related biomarkers, the 11 LASSO‐selected variables were further assessed using VIF and Spearman correlation analyses. CK, AST, and peak CK‐MB showed substantial collinearity or redundancy with other myocardial injury markers and were therefore excluded according to the predefined criteria (VIF > 5 or |*r*| > 0.70) (Figures 3 and 4). By contrast, although IL‐6 and hs‐CRP are biologically related inflammatory markers, their Spearman correlation coefficient was 0.41, which was below the prespecified exclusion threshold of 0.70. Their VIF values were 1.30 and 1.29, respectively; therefore, both variables were retained in the model. Individual ROC curves for the LASSO‐selected variables are shown in Figure 5. Because admission cTnI and peak cTnI represented closely related measures of myocardial injury, only one troponin variable was retained to avoid redundancy. Considering the intended use of the model for the early identification of patients at high risk of ACO, admission cTnI was retained in the model because of its earlier clinical availability. Ultimately, seven variables were included in the model: smoking history, STD ≥ 1 mm, RWMA, LVEF, admission cTnI, IL‐6, and hs‐CRP.

**Figure 2:**
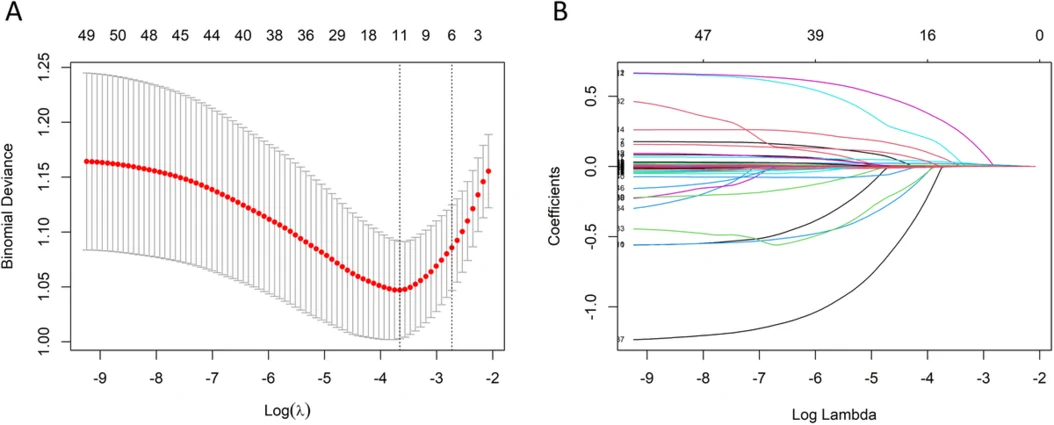
Variable selection using the LASSO regression: cross‐validation curve (A) and coefficient path (B). LASSO, least absolute shrinkage and selection operator.

**Figure 3:**
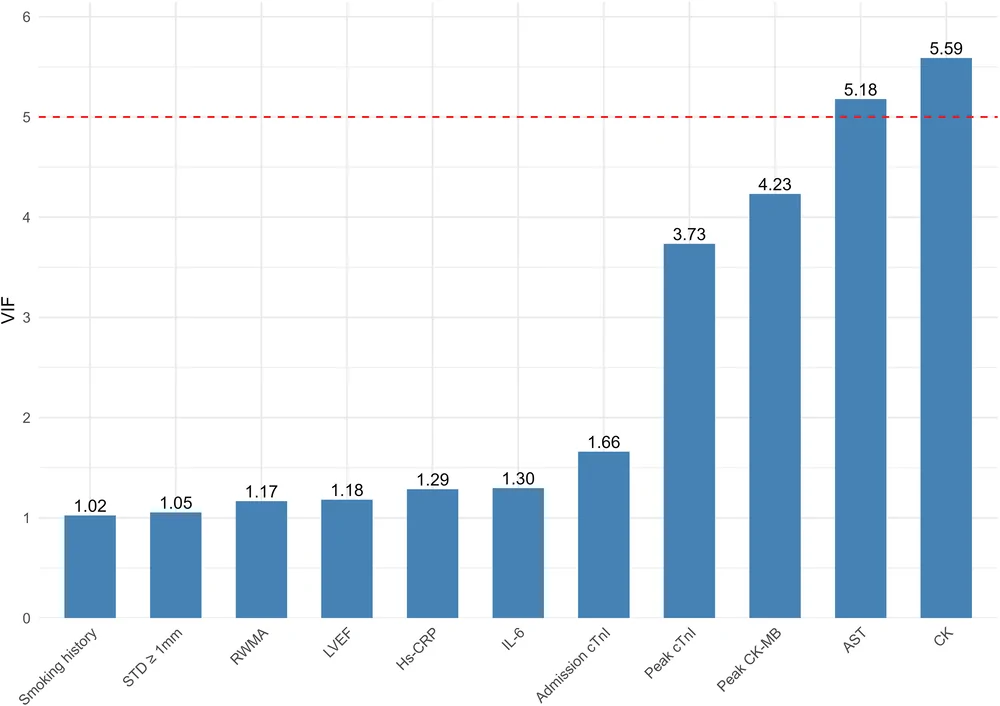
VIF analysis of variables selected by LASSO regression. AST, aspartate aminotransferase; CK, creatine kinase; cTnI, cardiac troponin I; Hs‐CRP, high‐sensitivity C‐reactive protein; IL‐6, interleukin‐6; LASSO, least absolute shrinkage and selection operator; LVEF, left ventricular ejection fraction; RWMA, regional wall motion abnormality; STD, ST‐segment depression; VIF, variance inflation factor.

**Figure 4:**
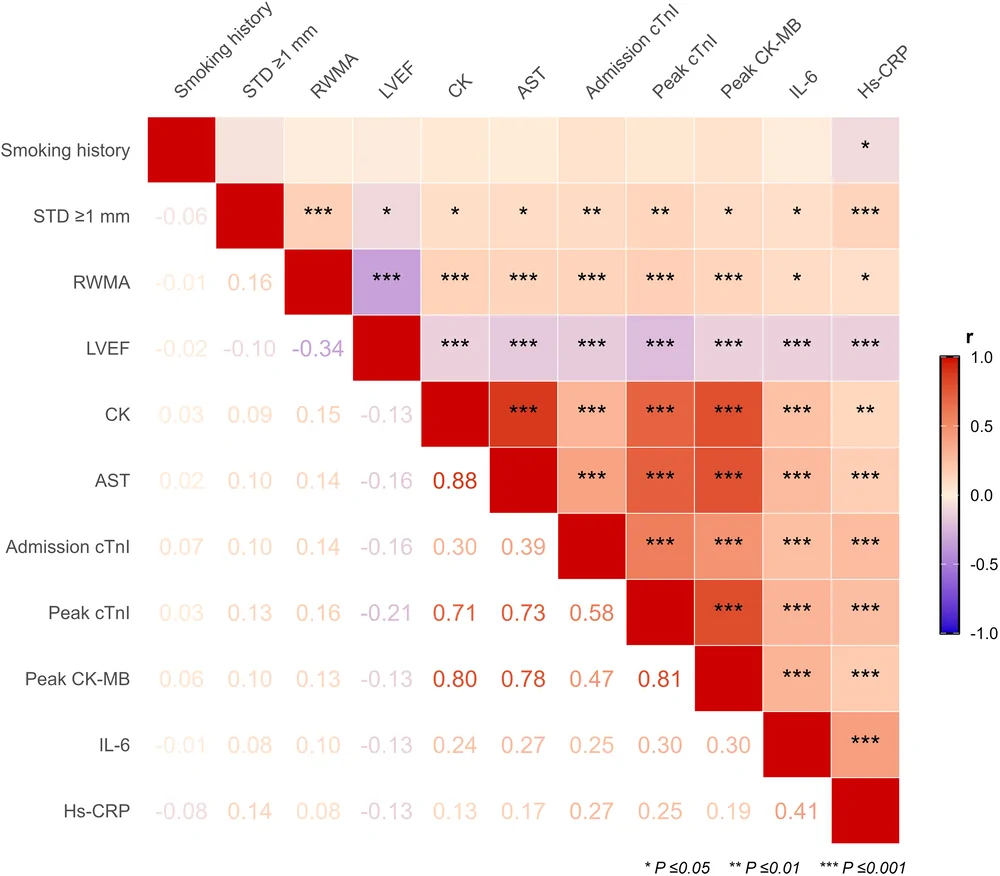
Correlation heatmap of variables selected by LASSO regression. AST, aspartate aminotransferase; CK, creatine kinase; cTnI, cardiac troponin I; Hs‐CRP, high‐sensitivity C‐reactive protein; IL‐6, interleukin‐6; LASSO, least absolute shrinkage and selection operator; LVEF, left ventricular ejection fraction; RWMA, regional wall motion abnormality; STD, ST‐segment depression.

**Figure 5:**
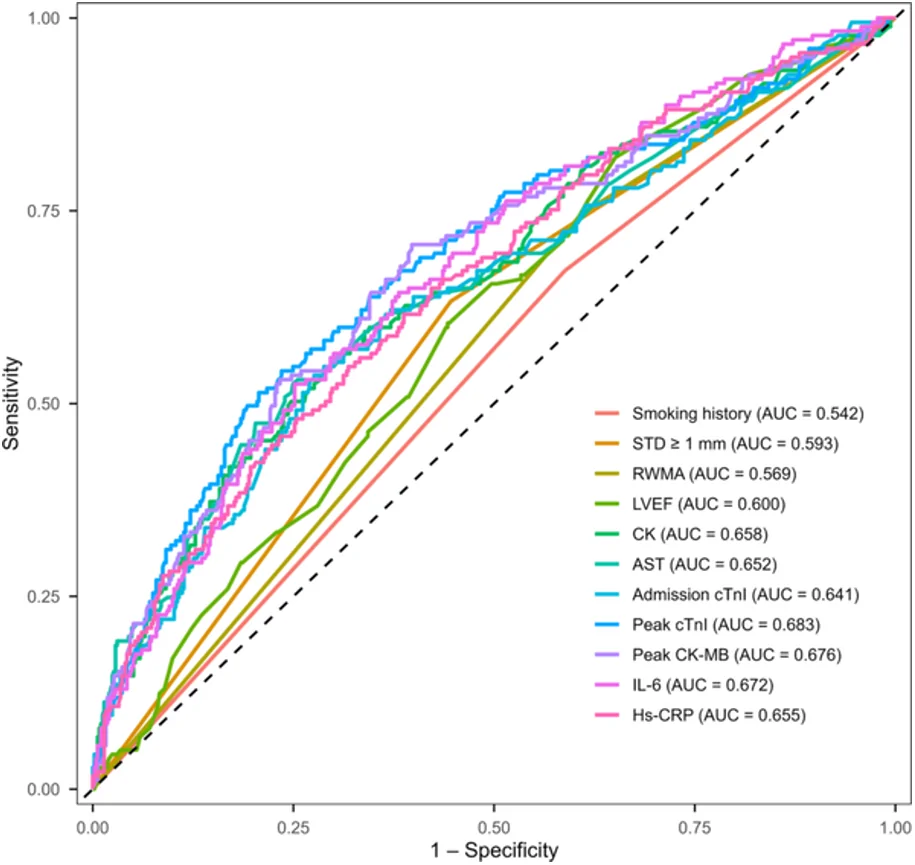
ROC curves of variables selected by LASSO regression for predicting acute coronary occlusion in patients with NSTEMI. AST, aspartate aminotransferase; AUC, area under the curve; CK, creatine kinase; CK‐MB, creatine kinase‐MB isoenzyme; cTnI, cardiac troponin I; Hs‐CRP, high‐sensitivity C‐reactive protein; IL‐6, interleukin‐6; LASSO, least absolute shrinkage and selection operator; LVEF, left ventricular ejection fraction; NSTEMI, non‐ST‐segment elevation myocardial infarction; ROC, receiver operating characteristic; RWMA, regional wall motion abnormality; STD, ST‐segment depression.

### 3.3 Multivariable Logistic Regression and Nomogram Construction

Smoking history, STD ≥ 1 mm, RWMA, LVEF, admission cTnI, IL‐6, and hs‐CRP were entered into the multivariable logistic regression analysis (Table 2). These variables were subsequently used to construct a nomogram for estimating the probability of ACO in NSTEMI patients (Figure 6). To facilitate clinical application, an interactive web‐based calculator was also developed (Supporting Information S1: Figure 1), which allows clinicians to input patient‐specific values for the seven predictors and obtain individualized risk estimates with 95% confidence intervals. The calculator is available at https://qichengyu.shinyapps.io/ACO_Calculator/.

**Table 2:** Multivariable logistic regression results in the training cohort.

Variables	β	SE	*Z*	*p*	OR (95% CI)
Smoking history	0.478	0.203	2.35	0.019	1.61 (1.08–2.40)
STD ≥ 1 mm	0.588	0.195	3.02	0.003	1.80 (1.23–2.64)
RWMA	0.297	0.220	1.35	0.177	1.35 (0.87–2.07)
LVEF	−0.014	0.011	−1.29	0.198	0.99 (0.96–1.01)
Admission cTnI	0.091	0.036	2.52	0.012	1.10 (1.02–1.18)
IL‐6	0.012	0.003	3.66	<0.001	1.01 (1.01–1.02)
Hs‐CRP	0.025	0.008	3.31	<0.001	1.03 (1.01–1.04)

Abbreviations: CI, confidence interval; Hs‐CRP, high‐sensitivity C‐reactive protein; IL‐6, interleukin‐6; LVEF, left ventricular ejection fraction; OR, odds ratio; RWMA, regional wall motion abnormality; STD, ST‐segment depression.

**Figure 6:**
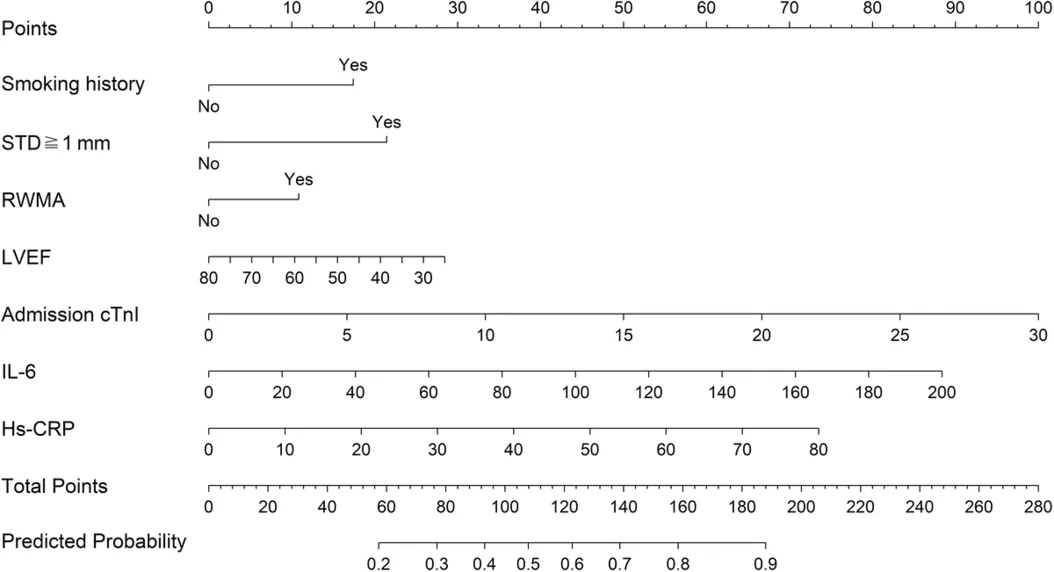
Nomogram for predicting ACO in patients with NSTEMI. Each predictor is assigned a score, and the total score corresponds to the estimated probability of ACO. ACO, acute coronary occlusion; cTnI, cardiac troponin I; IL‐6, interleukin‐6; Hs‐CRP, high‐sensitivity C‐reactive protein; LVEF, left ventricular ejection fraction; RWMA, regional wall motion abnormality; STD, ST‐segment depression.

### 3.4 Model Performance and Validation

The model demonstrated acceptable discrimination, with an AUC of 0.731 (95% CI, 0.688−0.773) in the training cohort and 0.715 (95% CI, 0.654−0.776) in the validation cohort (Figure 7). Measures of accuracy, sensitivity, specificity, PPV, and NPV are provided in Supporting Information S1: Table 6. Calibration plots demonstrated good agreement between predicted and observed outcomes, with Hosmer‐Lemeshow test *p* values of 0.233 and 0.200 for the training and validation cohorts, respectively (Figure 8). DCA showed favorable net clinical benefit across a range of threshold probabilities (Figure 9). Bootstrap internal validation showed limited optimism in model performance (Supporting Information S1: Table 7). The apparent AUC in the training cohort was 0.731, and the optimism‐corrected AUC was 0.717, with an estimated optimism of 0.014. The optimism‐corrected calibration slope was 0.926, and the optimism‐corrected Brier score was 0.172. To assess whether retention of RWMA and LVEF materially affected model performance, we performed a sensitivity analysis using a reduced model excluding both variables. The reduced model yielded slightly lower AUCs in the training cohort (0.727 vs. 0.731; ΔAUC = 0.004; DeLong *p* = 0.566) and the validation cohort (0.700 vs. 0.715; ΔAUC = 0.015; DeLong *p* = 0.239), but the differences were not statistically significant (Supporting Information S1: Table 8). Calibration plots showed similar agreement between predicted and observed probabilities for the primary and reduced models in both cohorts (Supporting Information S1: Figure 2). Given that IL‐6 is not routinely measured in all centers during the acute management of myocardial infarction, we further developed a simplified model excluding IL‐6 (Supporting Information S1: Table 9). In the training cohort, adding IL‐6 increased the AUC from 0.716 to 0.731, but the difference was not statistically significant (ΔAUC = 0.015, DeLong *p* = 0.122). In the validation cohort, the AUC was similar between the primary model and the simplified model (0.715 vs. 0.713; ΔAUC = 0.002, DeLong *p* = 0.733). In addition, an exploratory model replacing admission cTnI with peak cTnI was evaluated and is presented in Supporting Information S1: Table 10. This model yielded an AUC of 0.743 in the training cohort and 0.727 in the validation cohort. However, because peak cTnI is often unavailable during early clinical decision‐making, admission cTnI was retained in the model.

**Figure 7:**
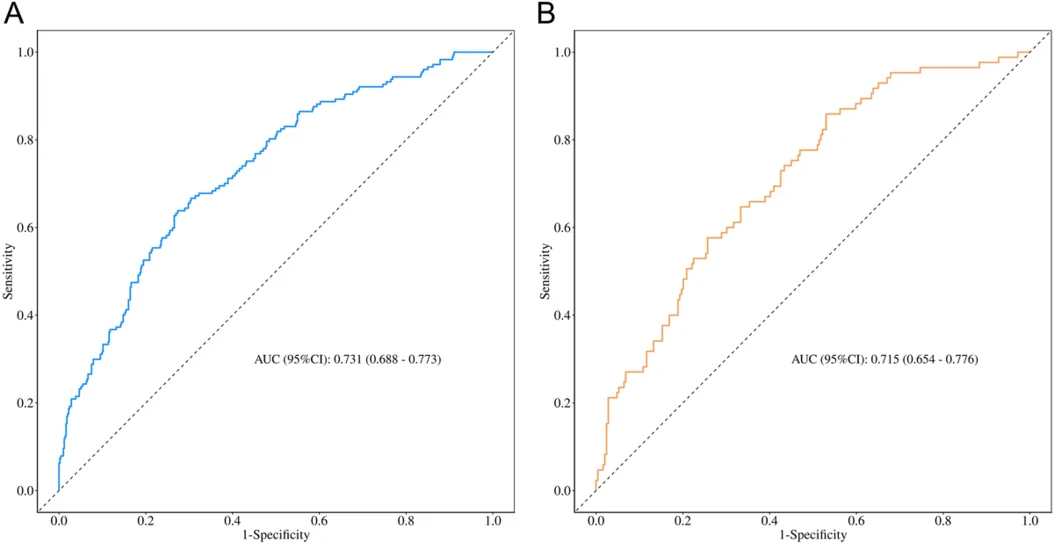
ROC curves for the prediction model in the training cohort (A) and validation cohort (B). ROC, receiver operating characteristic.

**Figure 8:**
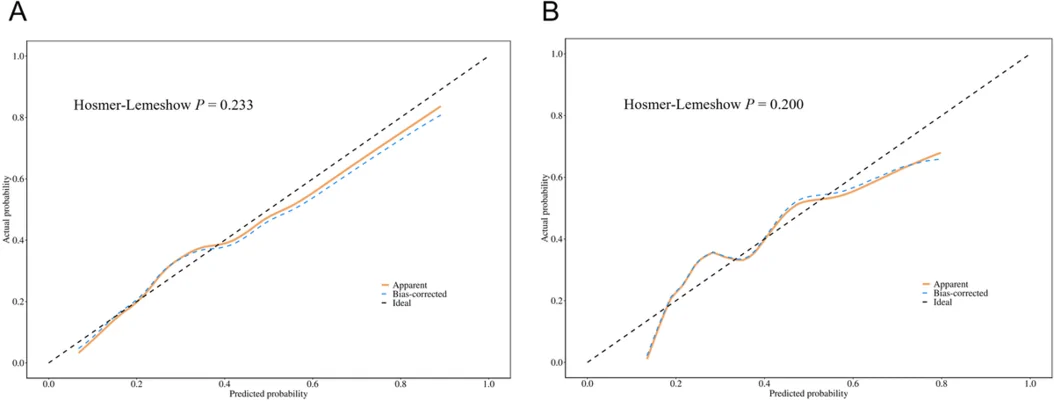
Calibration plots for the prediction model in the training cohort (A) and validation cohort (B).

**Figure 9:**
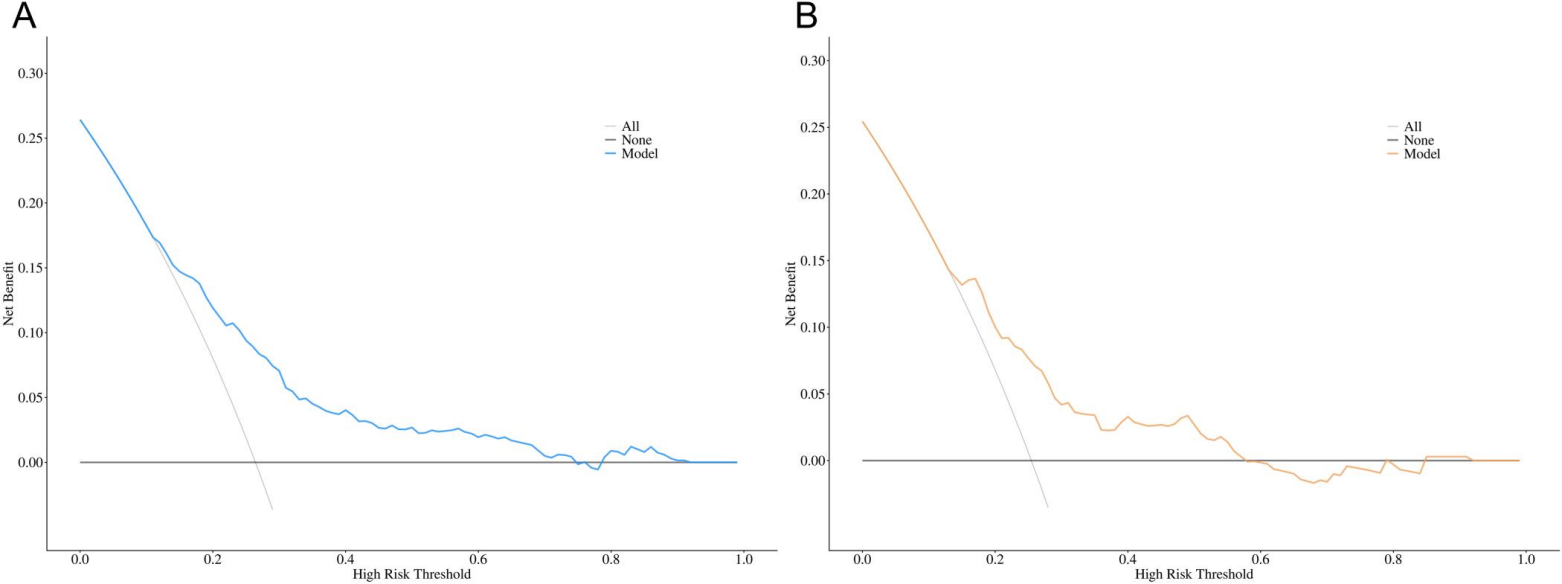
DCA for the prediction model in the training cohort (A) and validation cohort (B). DCA, decision curve analysis.

## 4 Discussion

This study found that approximately one‐quarter of NSTEMI patients presented with ACO, a prevalence consistent with prior research [12, 13], underscoring that this condition is not uncommon in clinical practice and has important clinical implications. We developed and temporally validated a nomogram based on multidimensional clinical indicators available during early hospitalization to estimate the probability of ACO in NSTEMI. The model showed acceptable discrimination, satisfactory calibration, and potential clinical utility. These findings suggest that the model may serve as an adjunctive tool for the early identification of patients at high risk of ACO who may warrant closer monitoring and prioritized invasive evaluation before coronary angiography.

In this study, the proportion of smoking history was higher in the ACO group, consistent with the findings of Ayad et al [27]. Mechanistically, smoking has been shown to induce endothelial dysfunction, promote platelet activation, and enhance a prothrombotic state, which may increase thrombus burden after plaque rupture and predispose to complete occlusion [28]. Previous studies have also reported a strong association between STD and the occurrence of OMI in NSTEMI [29, 30]. In addition, when maximum STD is localized to leads V1–V4, its specificity for posterior wall occlusion can reach 97% [31]. In our cohort, the prevalence of STD ≥ 1 mm was significantly higher in the ACO group than in the non‐ACO group, and this variable was retained in the prediction model by LASSO regression. However, its discriminatory capacity was limited, with an AUC of 0.593, indicating insufficient diagnostic utility when used in isolation. Recent studies showed that machine learning‐based ECG algorithms have achieved promising performance in detecting OMI [17, 18]. Future work may integrate AI‐ECG with clinical data and biomarkers to construct multimodal models, thereby enhancing the early identification of occlusive NSTEMI.

In NSTEMI patients, the presence and extent of RWMA are strongly associated with ACO [32], reflecting more severe localized ischemia and impaired contractile function. Notably, speckle‐tracking echocardiography‐derived regional circumferential strain has also shown potential for earlier identification of ACO in this population [15, 16]. In addition, a meta‐analysis reported that NSTEMI patients with ACO had lower LVEF, greater myocardial necrotic burden, and worse clinical outcomes compared with those without occlusion [13]. Consistent with these findings, our study demonstrated a higher prevalence of RWMA and significantly lower LVEF in the ACO group. RWMA reflects regional contractile impairment due to acute ischemia, whereas LVEF captures global ventricular dysfunction. In the subsequently fitted multivariable logistic regression model, neither RWMA nor LVEF reached statistical significance in the individual Wald tests. However, this model was used to estimate predictor coefficients and construct the nomogram, rather than to conduct an additional P value–based variable‐selection step. Accordingly, both variables were retained in the final model in accordance with the prespecified LASSO‐based selection strategy.

In this study, multiple myocardial injury biomarkers were significantly higher in the ACO group than in the non‐ACO group, indicating a greater burden of myocardial necrosis. This finding is consistent with prior evidence [13, 15, 33] and may be attributable to increased thrombus burden and sustained ischemia associated with complete coronary occlusion. In terms of inflammatory response, levels of inflammatory markers were also higher in the ACO group, in agreement with the observations of Bruno et al. [34]. To our knowledge, this study is among the first to quantitatively assess IL‐6 differences between ACO and non‐ACO patients, providing further support for the pre‐angiographic identification of occlusive NSTEMI. IL‐6 was incorporated into the model because it was routinely measured in our center and was selected by LASSO, supporting the potential contribution of inflammatory activation to ACO. However, we acknowledge that IL‐6 is not routinely measured in all centers for patients with AMI. Therefore, we additionally evaluated a simplified model excluding IL‐6, which showed comparable discrimination in the validation cohort. This simplified model may be more applicable in settings where IL‐6 is not readily available. These findings support the potential value of a multidimensional, clinically derived model for the early recognition of occlusive NSTEMI.

One important limitation of this study is that some variables were collected not during the emergency visit but within the first 24 h of hospitalization. The current guideline recommends an early invasive strategy for NSTEMI patients, generally within 24 h of admission and up to 72 h in selected intermediate‐risk patients [11], which supports the rationale of our study design. By contrast, STEMI care targets immediate reperfusion, with a first medical contact to device time of ≤ 90 min at PCI‐capable centers (≤ 120 min with interhospital transfer) and reperfusion within 12 h of symptom onset [11]. Hence, the 24‐h window may still result in delayed revascularization for NSTEMI patients with ACO. Notably, prior studies have demonstrated that STEMI patients may still benefit from revascularization performed 12−48 h after symptom onset [35, 36], and this benefit may also extend to NSTEMI patients with ACO. In clinical practice, the proportion of NSTEMI patients who receive early revascularization as recommended by guidelines remains suboptimal [37]. Therefore, the model may help clinicians identify patients at higher risk of coronary occlusion while awaiting invasive evaluation, support closer monitoring and earlier involvement of the interventional team, and facilitate prioritization of invasive evaluation within 24 h when clinically feasible. Future research should aim to construct and validate prediction models for OMI based on features obtainable in the emergency setting.

Although archived ECG tracings were re‐reviewed by senior attending cardiologists blinded to coronary angiography findings, most tracings were obtained from photographed or digitally archived records, and image quality was occasionally suboptimal. Therefore, minor measurement error or misclassification of the extent and lead distribution of STD cannot be completely excluded. In addition, although standard paper speed and voltage calibration were used for ECG acquisition, ECG machine models were not completely consistent over the long study period. Although STD was further classified by lead distribution, ischemic STD could not be fully distinguished from non‐ischemic repolarization abnormalities, such as left ventricular hypertrophy‐related strain patterns, conduction abnormalities, or other baseline repolarization changes, which may have attenuated its observed association with ACO.

Pre‐admission medication exposure, particularly prior statin therapy, was not systematically collected because of the inherent uncertainty and incompleteness of medication records in the retrospective electronic medical records. Therefore, these variables could not be reliably incorporated into the prediction model. This represents a limitation of the present study, as prior statin therapy and other cardiovascular medications may influence plaque morphology, thrombotic burden, and the likelihood of occlusive versus non‐occlusive infarction. Future prospective studies should systematically collect pre‐admission medication use, including treatment duration, adherence, and statin intensity, to further evaluate its potential impact on ACO risk prediction.

In addition, this was a single‐center retrospective study, which is subject to the inherent risk of bias associated with retrospective designs. Although temporal validation was performed using a later cohort from the same institution, this approach does not constitute true external validation and cannot fully establish generalizability across different hospitals, patient populations, laboratory platforms, or clinical workflows. Therefore, independent multicenter external validation is required before broad clinical implementation. Future studies should focus on establishing large, multicenter prospective cohorts and integrating advanced technologies such as speckle‐tracking echocardiography and AI‐ECG to further improve the model's predictive accuracy and clinical applicability.

## 5 Conclusion

In summary, the prediction model for ACO in patients with NSTEMI, developed from multidimensional clinical variables, showed acceptable discrimination, satisfactory calibration, and potential clinical utility. It may help identify patients at high risk of ACO before coronary angiography and support the prioritization of invasive evaluation when clinically feasible.

## Author Contributions

**Qicheng Yu:** conceptualization, methodology, validation, formal analysis, data curation, writing – original draft. **Shuai Wang:** investigation, data curation. **Yuanyuan Zhao:** investigation, data curation. **Yanlong Zhao:** investigation, data curation. **Shan Xie:** investigation, data curation. **Yanling Wang:** investigation, project administration, supervision. **Zhenxing Fan:** investigation, project administration, supervision. **Fangyan Liu:** investigation, supervision. **Zhi Liu:** resources, funding acquisition, project administration, supervision, writing – review and editing.

## Ethics Statement

The study protocol was approved by the Ethics Committee of Xuanwu Hospital, Capital Medical University (Approval No. KS2022161‐1).

## Conflicts of Interest

The authors declare no conflicts of interest.

## Supporting information


Supporting File

## Data Availability

The data underlying this article will be shared on reasonable request to the corresponding author.
