# Supplementary material for: Development and Validation of a Prediction Model for Acute Coronary Occlusion in Patients With Non‐ST‐Segment Elevation Myocardial Infarction
Source: Clin Cardiol. 2026 Oct 22;49(10):e70492. doi: 10.1002/clc.70492 (PMC13688425; doi:10.1002/clc.70492)
Supplement: Supplementary file 1 [file CLC-49-e70492-s001.docx]

**Supplementary Appendix** Candidate variables initially included in the LASSO regression

The following 50 variables were initially included in the LASSO regression:

**Demographics and clinical characteristics:** Sex, Age, BMI, HR, SBP, DBP, Killip class ≥2, Hypertension, DM, Previous MI, Smoking history.

**Electrocardiography:** ST-segment depression ≥1 mm, T-wave inversion.

**Echocardiography:** RWMA, LVEF.

**Laboratory tests:** Myoglobin, LDH, α-HBDH, AST, CK, admission CK-MB, admission cTnI, peak CK-MB, peak cTnI, NT-proBNP, IL-6, hs-CRP, creatinine, eGFR, uric acid, Hcy, HDL-C, LDL-C, Apolipoprotein A1, Apolipoprotein B, TG, TC, SHR, TSH, T3, FT3, FT4, Fibrinogen, D-dimer, White blood cell count, Monocyte count, Neutrophil-to-lymphocyte ratio, Hemoglobin, Albumin, Platelet count.

**Abbreviations:** HR, heart rate; SBP, systolic blood pressure; DBP, diastolic blood pressure; BMI, body mass index; DM, diabetes mellitus; MI, myocardial infarction; RWMA, regional wall motion abnormality; LVEF, left ventricular ejection fraction; LDH, lactate dehydrogenase; α-HBDH, α-hydroxybutyrate dehydrogenase; AST, aspartate aminotransferase; CK, creatine kinase; CK-MB, creatine kinase-MB; cTnI, cardiac troponin I; NT-proBNP, N-terminal pro-B-type natriuretic peptide; IL-6, interleukin-6; hs-CRP, high-sensitivity C-reactive protein; eGFR, estimated glomerular filtration rate; Hcy, homocysteine; HDL-C, high-density lipoprotein cholesterol; LDL-C, low-density lipoprotein cholesterol; TG, triglycerides; TC, total cholesterol; SHR, stress hyperglycemia ratio; TSH, thyroid-stimulating hormone; T3, triiodothyronine; FT3, free triiodothyronine; FT4, free thyroxine.

**Supplementary Table 1** Missing data summary in the training cohort

| Variable | N | Missing (n) | Missing Percentage |
| --- | --- | --- | --- |
| CK | 670 | 6 | 0.8% |
| Myoglobin | 670 | 10 | 1.4% |
| α-HBDH | 670 | 9 | 1.3% |
| LDH | 670 | 9 | 1.3% |
| AST | 670 | 2 | 0.2% |
| NT-proBNP | 670 | 47 | 7.0% |
| IL-6 | 670 | 61 | 9.1% |
| Hs-CRP | 670 | 100 | 14.9% |
| Albumin | 670 | 4 | 0.5% |
| Uric acid | 670 | 3 | 0.4% |
| HDL-C | 670 | 5 | 0.7% |
| LDL-C | 670 | 4 | 0.5% |
| Apolipoprotein A1 | 670 | 9 | 1.3% |
| Apolipoprotein B | 670 | 9 | 1.3% |
| Triglyceride | 670 | 3 | 0.4% |
| Total cholesterol | 670 | 3 | 0.4% |
| Hcy | 670 | 33 | 4.9% |
| Glycated hemoglobin | 670 | 34 | 5.0% |
| TSH | 670 | 17 | 2.5% |
| T3 | 670 | 17 | 2.5% |
| FT3 | 670 | 17 | 2.5% |
| FT4 | 670 | 17 | 2.5% |
| White blood cell | 670 | 4 | 0.5% |
| Monocyte | 670 | 4 | 0.5% |
| Neutrophil | 670 | 4 | 0.5% |
| Lymphocyte | 670 | 4 | 0.5% |
| Hemoglobin | 670 | 4 | 0.5% |
| Platelet | 670 | 4 | 0.5% |
| Fibrinogen | 670 | 17 | 2.5% |
| D-dimer | 670 | 27 | 4.0% |

**Supplementary Table 2** Missing data summary in the validation cohort

| Variable | N | Missing (n) | Missing Percentage |
| --- | --- | --- | --- |
| CK | 334 | 2 | 0.5% |
| Myoglobin | 334 | 2 | 0.5% |
| α-HBDH | 334 | 2 | 0.5% |
| LDH | 334 | 2 | 0.5% |
| NT-proBNP | 334 | 18 | 5.3% |
| IL-6 | 334 | 65 | 19.4% |
| Hs-CRP | 334 | 33 | 9.8% |
| Albumin | 334 | 2 | 0.5% |
| Apolipoprotein A1 | 334 | 2 | 0.5% |
| Apolipoprotein B | 334 | 2 | 0.5% |
| Hcy | 334 | 44 | 13.1% |
| Glycated hemoglobin | 334 | 35 | 10.4% |
| TSH | 334 | 10 | 2.9% |
| T3 | 334 | 10 | 2.9% |
| FT3 | 334 | 10 | 2.9% |
| FT4 | 334 | 10 | 2.9% |
| Fibrinogen | 334 | 3 | 0.8% |
| D-dimer | 334 | 3 | 0.8% |

**Supplementary Table 3** Clinical characteristics of patients in the training and validation cohorts.

| Variables | Total  (n = 1004) | Training  (n = 670) | Validation  (n = 334) | *P* |
| --- | --- | --- | --- | --- |
|  |  |  |  |  |
| ACO, n (%) | 262 (26.1) | 177 (26.4) | 85 (25.4) | 0.742 |
| Age (years) | 63.00 (56.00, 70.00) | 62.00 (56.00, 69.75) | 63.00 (56.25, 70.00) | 0.908 |
| Sex (Male), n (%) | 784 (78.1) | 516 (77.0) | 268 (80.2) | 0.244 |
| Heart rate (bpm) | 74.00 (66.00, 83.00) | 74.00 (66.00, 83.00) | 74.00 (67.00, 83.50) | 0.544 |
| BMI (kg/m^2^) | 25.71 (23.81, 27.76) | 25.76 (23.94, 27.77) | 25.40 (23.46, 27.68) | 0.286 |
| SBP (mmHg) | 133.00 (121.00, 146.00) | 134.00 (122.00, 147.75) | 130.00 (119.25, 144.00) | 0.025 |
| DBP (mmHg) | 74.00 (66.00, 83.00) | 75.00 (67.00, 84.00) | 74.00 (66.00, 80.00) | 0.011 |
| Smoking history, n (%) | 593 (59.1) | 409 (61.0) | 184 (55.1) | 0.071 |
| Alcohol use, n (%) | 389 (38.7) | 268 (40.0) | 121 (36.2) | 0.248 |
| Hypertension, n (%) | 644 (64.1) | 438 (65.4) | 206 (61.7) | 0.250 |
| DM, n (%) | 392 (39.0) | 273 (40.7) | 119 (35.6) | 0.117 |
| Previous MI, n (%) | 120 (12.0) | 60 (9.0) | 60 (18.0) | <0.001 |
| Previous PCI, n (%) | 145 (14.4) | 76 (11.3) | 69 (20.7) | <0.001 |
| Previous stroke, n (%) | 144 (14.3) | 92 (13.7) | 52 (15.6) | 0.434 |
| CK (IU/L) | 135.50 (80.00, 326.25) | 148.50 (83.25, 333.50) | 125.00 (76.00, 288.50) | 0.057 |
| Myoglobin (ng/mL) | 48.00 (22.53, 110.25) | 44.00 (22.10, 96.30) | 59.55 (23.77, 128.00) | 0.008 |
| α-HBDH (IU/L) | 178.00 (136.00, 259.00) | 181.00 (141.00, 269.00) | 162.00 (126.00, 239.00) | <0.001 |
| LDH (IU/L) | 218.50 (175.00, 300.50) | 221.00 (178.00, 305.00) | 210.50 (166.00, 291.50) | 0.056 |
| AST (IU/L) | 30.00 (22.00, 52.00) | 31.00 (22.00, 55.00) | 28.00 (21.00, 45.75) | 0.020 |
| Admission CK-MB (ng/mL) | 8.10 (3.30, 23.00) | 8.54 (3.25, 25.00) | 7.31 (3.60, 18.85) | 0.272 |
| Admission cTnI (ng/mL) | 0.28 (0.09, 0.98) | 0.29 (0.09, 1.10) | 0.24 (0.07, 0.76) | 0.070 |
| NT-proBNP (pg/mL) | 473.50 (175.00, 1219.75) | 500.00 (189.00, 1242.25) | 415.00 (150.00, 1170.00) | 0.149 |
| Peak CK-MB (ng/mL) | 17.00 (5.56, 57.70) | 16.65 (5.13, 58.00) | 17.75 (6.72, 56.22) | 0.263 |
| Peak cTnI (ng/mL) | 1.16 (0.31, 4.19) | 1.12 (0.31, 4.36) | 1.21 (0.35, 3.53) | 0.862 |
| IL-6 (pg/mL) | 17.23 (9.06, 30.78) | 19.16 (9.42, 32.82) | 14.57 (8.48, 23.38) | <0.001 |
| Hs-CRP (mg/L) | 6.99 (2.29, 17.31) | 6.93 (2.30, 16.17) | 7.29 (2.29, 18.77) | 0.688 |
| Albumin (g/L) | 37.16 (35.00, 39.40) | 37.14 (34.96, 39.40) | 37.21 (35.13, 39.48) | 0.612 |
| eGFR (mL/min/1.73 m^2^) | 99.37 (89.60, 106.15) | 100.19 (90.56, 106.16) | 98.00 (86.80, 105.89) | 0.099 |
| Creatinine (μmol/L) | 71.00 (61.00, 82.00) | 70.00 (61.00, 81.00) | 72.00 (62.00, 86.00) | 0.067 |
| Uric acid (μmol/L) | 343.00 (287.00, 414.00) | 340.00 (279.25, 403.75) | 354.00 (296.25, 426.00) | 0.016 |
| HDL-C (mmol/L) | 0.97 (0.84, 1.15) | 0.98 (0.85, 1.17) | 0.96 (0.81, 1.11) | 0.016 |
| LDL-C (mmol/L) | 2.54 (1.99, 3.10) | 2.54 (2.04, 3.09) | 2.51 (1.94, 3.13) | 0.571 |
| Apolipoprotein A1 (g/L) | 1.09 (0.98, 1.22) | 1.09 (0.97, 1.22) | 1.10 (0.99, 1.23) | 0.365 |
| Apolipoprotein B (g/L) | 0.89 (0.75, 1.06) | 0.87 (0.74, 1.04) | 0.92 (0.76, 1.08) | 0.005 |
| Triglyceride (mmol/L) | 1.63 (1.17, 2.28) | 1.65 (1.16, 2.31) | 1.58 (1.17, 2.26) | 0.774 |
| Total cholesterol (mmol/L) | 4.20 (3.57, 4.90) | 4.17 (3.57, 4.88) | 4.28 (3.57, 4.93) | 0.336 |
| Hcy (μmol/L) | 14.40 (11.40, 18.13) | 14.62 (11.60, 18.40) | 14.08 (10.93, 17.72) | 0.039 |
| FBG (mmol/L) | 6.00 (5.07, 7.92) | 6.08 (5.06, 7.92) | 5.93 (5.11, 7.98) | 0.701 |
| Glycated hemoglobin (%) | 6.20 (5.60, 7.40) | 6.20 (5.62, 7.50) | 6.10 (5.60, 7.20) | 0.245 |
| SHR | 0.84 (0.75, 0.95) | 0.83 (0.74, 0.95) | 0.85 (0.76, 0.97) | 0.046 |
| TSH (μIU/mL) | 1.46 (0.92, 2.26) | 1.42 (0.90, 2.25) | 1.58 (0.99, 2.39) | 0.248 |
| T3 (ng/mL) | 0.94 (0.80, 1.06) | 0.94 (0.80, 1.04) | 0.94 (0.80, 1.09) | 0.451 |
| FT3 (pg/mL) | 2.74 (2.44, 2.99) | 2.66 (2.40, 2.91) | 2.85 (2.57, 3.10) | <0.001 |
| FT4 (ng/dL) | 1.11 (1.00, 1.23) | 1.12 (1.01, 1.25) | 1.09 (0.98, 1.19) | 0.001 |
| NLR | 2.88 (2.09, 4.16) | 2.88 (2.10, 4.06) | 2.85 (2.09, 4.33) | 0.644 |
| White blood cells (×10^9^/L) | 7.98 (6.57, 9.56) | 8.02 (6.67, 9.70) | 7.80 (6.38, 9.27) | 0.242 |
| Monocytes (×10^9^/L) | 0.48 (0.37, 0.61) | 0.46 (0.36, 0.59) | 0.51 (0.40, 0.64) | <0.001 |
| Neutrophils (×10^9^/L) | 5.37 (4.21, 6.74) | 5.46 (4.26, 6.75) | 5.15 (4.11, 6.73) | 0.150 |
| Lymphocytes (×10^9^/L) | 1.84 (1.41, 2.31) | 1.88 (1.44, 2.34) | 1.76 (1.36, 2.24) | 0.037 |
| Hemoglobin (g/L) | 137.00 (125.00, 147.00) | 138.00 (126.00, 147.00) | 135.00 (123.00, 146.75) | 0.058 |
| Platelets (×10^9^/L) | 212.00 (175.00, 252.00) | 215.00 (175.00, 257.75) | 204.00 (173.50, 241.75) | 0.023 |
| Fibrinogen (g/L) | 3.55 (3.04, 4.21) | 3.55 (3.05, 4.16) | 3.55 (3.02, 4.55) | 0.321 |
| D-dimer (μg/mL) | 0.30 (0.20, 0.46) | 0.30 (0.21, 0.47) | 0.27 (0.19, 0.44) | 0.022 |
| LVEF (%) | 60.0 (55.0, 66.0) | 61.0 (55.0, 67.0) | 59.5 (53.0, 64.0) | <0.001 |
| RWMA, n (%) | 637 (63.4) | 432 (64.5) | 205 (61.4) | 0.336 |
| Killip≥2, n (%) | 275 (27.4) | 194 (29.0) | 81 (24.3) | 0.115 |
| GRACE score (points) | 133.00 (112.00, 153.00) | 136.00 (113.00, 155.00) | 129.00 (109.25, 149.00) | 0.012 |
| ST-segment depression ≥1 mm, n (%) | 418 (41.6) | 332 (49.6) | 86 (25.7) | <0.001 |
| T wave inversion, n (%) | 372 (37.1) | 264 (39.4) | 108 (32.3) | 0.029 |

ACO, acute coronary occlusion; AST, aspartate aminotransferase; α-HBDH, α-hydroxybutyrate dehydrogenase; BMI, body mass index; CK, creatine kinase; CK-MB, creatine kinase-MB isoenzyme; cTnI, cardiac troponin I; DBP, diastolic blood pressure; DM, diabetes mellitus; eGFR, estimated glomerular filtration rate; FBG, fasting blood glucose; FT3, free triiodothyronine; FT4, free thyroxine; GRACE, Global Registry of Acute Coronary Events; Hcy, homocysteine; HDL-C, high-density lipoprotein cholesterol; Hs-CRP, high-sensitivity C-reactive protein; IL-6, interleukin-6; LDH, lactate dehydrogenase; LDL-C, low-density lipoprotein cholesterol; LVEF, left ventricular ejection fraction; MI, myocardial infarction; NLR, neutrophil-to-lymphocyte ratio; NT-proBNP, N-terminal pro-B-type natriuretic peptide; PCI, percutaneous coronary intervention; RWMA, regional wall motion abnormality; SBP, systolic blood pressure; SHR, stress hyperglycemia ratio; T3, triiodothyronine; TSH, thyroid-stimulating hormone.

**Supplementary Table 4** Lead-distribution patterns of ST-segment depression in patients with and without ACO

| **ECG pattern** | **Non-ACO** | **ACO** | ***P*** |
| --- | --- | --- | --- |
| Any STD ≥1 mm | 220 (44.6%) | 112 (63.3%) | <0.001 |
| Anterior precordial STD, V1–V4 | 187 (37.9%) | 95 (53.7%) | <0.001 |
| Inferior STD, II/III/aVF | 60 (12.2%) | 34 (19.2%) | 0.021 |
| Lateral STD, I/aVL/V5–V6 | 184 (37.3%) | 90 (50.8%) | 0.002 |
| Multiterritory STD | 170 (34.5%) | 86 (48.6%) | <0.001 |

ACO, acute coronary occlusion; STD, ST-segment depression.

**Supplementary Table 5** Interobserver agreement for ST-segment depression ≥1 mm assessment

| **Assessment** | **N** | **Agreement, n (%)** | **Disagreement, n (%)** | **Cohen’s κ (95% CI)** | ***P*** |
| --- | --- | --- | --- | --- | --- |
| ST-segment depression ≥1 mm | 1004 | 964 (96.0) | 40 (4.0) | 0.918 (0.892–0.942) | <0.001 |

Agreement for ST-segment depression ≥1 mm was assessed between two independent ECG readers. Cohen’s κ was used to quantify interobserver agreement. CI, confidence interval.

**Supplementary Table 6** Discriminative performance of the prediction model in the training and validation cohorts

|  | AUC (95%CI) | Accuracy (95%CI) | Sensitivity (95%CI) | Specificity (95%CI) | PPV (95%CI) | NPV (95%CI) | cut off |
| --- | --- | --- | --- | --- | --- | --- | --- |
|  |  |  |  |  |  |  |  |
| Training | 0.731 (0.688-0.773) | 0.703 (0.667-0.737) | 0.638  (0.568 - 0.709) | 0.726  (0.687 - 0.766) | 0.456 (0.394 - 0.518) | 0.848 (0.814 - 0.883) | 0.262 |
| Validation | 0.715 (0.654-0.776) | 0.716 (0.664-0.763) | 0.447  (0.341 - 0.553) | 0.807  (0.758 - 0.856) | 0.442 (0.337 - 0.547) | 0.810 (0.762 - 0.859) | 0.262 |

AUC, area under the receiver operating characteristic curve; CI, confidence interval; NPV, negative predictive value; PPV, positive predictive value.

**Supplementary Table 7** Bootstrap-corrected performance of the primary prediction model

| Measure | Apparent  performance | Mean  optimism | Optimism-corrected  performance |
| --- | --- | --- | --- |
|  |  |  |  |
| AUC | 0.731 | 0.014 | 0.717 |
| Brier score | 0.167 | 0.005 | 0.172 |
| Calibration slope | 1.000 | 0.074 | 0.926 |
| Calibration intercept | 0.000 | -0.005 | 0.005 |

AUC, area under the receiver operating characteristic curve.

**Supplementary Table 8** Comparison of model discrimination with and without RWMA and LVEF

| Dataset | Model | AUC | 95% CI | ΔAUC | DeLong *P* |
| --- | --- | --- | --- | --- | --- |
| Training cohort | Reduced model | 0.727 | 0.684–0.770 | 0.004 | 0.566 |
|  | Primary model | 0.731 | 0.688–0.773 |  |  |
| Validation cohort | Reduced model | 0.700 | 0.638–0.763 | 0.015 | 0.239 |
|  | Primary model | 0.715 | 0.654–0.776 |  |  |

The primary model included smoking history, ST-segment depression ≥1 mm, RWMA, LVEF, admission cTnI, IL-6, and hs-CRP. The reduced model excluded RWMA and LVEF while retaining the other predictors. ΔAUC and DeLong P values indicate comparisons between the primary and reduced models within each cohort. AUC, area under the receiver operating characteristic curve; CI, confidence interval; cTnI, cardiac troponin I; hs-CRP, high-sensitivity C-reactive protein; IL-6, interleukin-6; LVEF, left ventricular ejection fraction; RWMA, regional wall motion abnormality.

**Supplementary Table 9** Comparison of model discrimination with and without IL-6

| Dataset | Model | AUC | 95% CI | ΔAUC | DeLong *P* |
| --- | --- | --- | --- | --- | --- |
| Training cohort | Simplified model | 0.716 | 0.672–0.759 | 0.015 | 0.122 |
|  | Primary model | 0.731 | 0.688–0.773 |  |  |
| Validation cohort | Simplified model | 0.713 | 0.652–0.773 | 0.002 | 0.733 |
|  | Primary model | 0.715 | 0.654–0.776 |  |  |

The primary model included smoking history, ST-segment depression ≥1 mm, RWMA, LVEF, admission cTnI, IL-6, and hs-CRP. The simplified model excluded IL-6 while retaining the other predictors. ΔAUC and DeLong *P* values indicate comparisons between the primary and simplified models within each cohort. AUC, area under the receiver operating characteristic curve; CI, confidence interval; cTnI, cardiac troponin I; hs-CRP, high-sensitivity C-reactive protein; IL-6, interleukin-6; LVEF, left ventricular ejection fraction; RWMA, regional wall motion abnormality.

**Supplementary Table 10** Comparison of model discrimination using admission cTnI and peak cTnI

| Model | Troponin variable | Training cohort  AUC (95% CI) | Validation cohort  AUC (95% CI) |
| --- | --- | --- | --- |
| Primary model | Admission cTnI | 0.731 (0.688-0.773) | 0.715 (0.654-0.776) |
| Exploratory model | Peak cTnI | 0.743 (0.701-0.785) | 0.727 (0.666-0.789) |

The exploratory model replaced admission cTnI with peak cTnI, while all other predictors were kept unchanged. AUC, area under the receiver operating characteristic curve; CI, confidence interval; cTnI, cardiac troponin I.

**Supplementary Figure 1** The interactive web-based calculator for predicting acute coronary occlusion (ACO) in patients with NSTEMI. The calculator was developed from the final multivariable logistic regression model and allows users to enter patient-specific values for the seven predictors: smoking history, ST-segment depression ≥1 mm, regional wall motion abnormality, left ventricular ejection fraction, admission cardiac troponin I, interleukin-6, and high-sensitivity C-reactive protein. The tool provides individualized estimates of ACO probability with 95% confidence intervals.

**Supplementary Figure 2** Calibration plots of the primary and reduced models in the training and validation cohorts. The solid lines with circles represent the primary model, whereas the dashed lines with triangles represent the reduced model excluding RWMA and LVEF. The diagonal dotted line indicates perfect calibration.
